# A Multimodal Approach Utilizing Balloon Occlusion for Postpancreatectomy Hemorrhage: A Case Report

**DOI:** 10.70352/scrj.cr.24-0014

**Published:** 2025-02-26

**Authors:** Aya Maekawa, Takafumi Sato, Satoshi Tsuchiya, Kosuke Kobayashi, Atsushi Oba, Yoshihiro Ono, Hiromichi Ito, Yosuke Inoue, Kiyoshi Matsueda, Yu Takahashi

**Affiliations:** 1 Division of Hepatobiliary and Pancreatic Surgery, Cancer Institute Hospital, Japanese Foundation for Cancer Research, Tokyo, Japan; 2 Department of Diagnostic Ultrasound and Interventional Radiology, Cancer Institute Hospital, Japanese Foundation for Cancer Research, Tokyo, Japan

**Keywords:** postpancreatectomy hemorrhage, interventional radiology, balloon occlusion

## Abstract

**INTRODUCTION:**

Postpancreatectomy hemorrhage (PPH) is a life-threatening complication following pancreaticoduodenectomy, requiring prompt and accurate diagnostic and therapeutic measures to ensure patient survival.

**CASE PRESENTATION:**

A 79-year-old man underwent robot-assisted pancreaticoduodenectomy for suspected intraductal papillary mucinous carcinoma. Postoperatively, he developed a pancreatic fistula and major bile leak, leading to a hemorrhagic event on postoperative day 6. Initial stabilization was achieved with intravenous fluids and blood transfusions, followed by emergent angiography, which identified bleeding from the gastroduodenal artery (GDA) stump. Due to the short length of the remaining GDA, simple embolization of the GDA stump was considered inadequate. Given the anatomy of the short proper hepatic artery (PHA) and its immediate bifurcation into the left and right hepatic arteries, coil embolization was feared to cause infarction of the entire liver, and even with stenting, the left hepatic artery (LHA) would have to be sacrificed. Temporary balloon occlusion of the common hepatic artery (CHA) was used to stabilize the hemodynamics, serving as a bridge to surgical intervention to maintain hepatic blood flow. Although it was an emergency laparotomy, intraoperative CHA balloon occlusion created a controlled environment, allowing for precise localization and effective management of the hemorrhage. The root of the GDA was ligated, and hepatic blood flow was preserved. The choledochojejunostomy leak was repaired by re-anastomosis. The patient was discharged following successful conservative management of the pancreatic fistula. Eight months post-intervention, follow-up imaging confirmed preserved hepatic arterial flow.

**CONCLUSION:**

This case underscores the efficacy of a multidisciplinary approach in managing delayed PPH in hemodynamically stable patients. Comprehensive angiographic assessment, combined with temporary CHA balloon occlusion for bleeding control and meticulous surgical hemostasis, offers a viable strategy ensuring immediate and mid-term patient well-being.

## Abbreviations

CA
: celiac axis
CHA
: common hepatic artery
CT
: computed tomography
GDA
: gastroduodenal artery
IR
: interventional radiology
LHA
: left hepatic artery
OR
: operating room
POD
: postoperative day
PPH
: postpancreatectomy hemorrhage
PSPDA
: posterior superior pancreaticoduodenal artery
RHA
: right hepatic artery

## INTRODUCTION

Postpancreatectomy hemorrhage (PPH) is a potentially fatal complication that can occur after pancreaticoduodenectomy.^1)^ Delayed PPH, which manifests more than 24 hours post-operation, presents significant challenges in both diagnosis and management.^2)^

Treatment options for PPH are often guided by the patient’s hemodynamic stability and findings from computed tomography (CT) imaging, with interventional radiology (IR) playing a prominent role in management.^3)^ While embolization and stenting are considered less invasive and effective options, they carry the risk of arterial occlusion, potentially leading to severe hepatic complications such as abscess formation and liver failure.^4–6)^ Conversely, laparotomy offers the advantage of addressing the underlying causes of delayed PPH, providing hemostatic options, including the use of hemostatic materials, sutures, ligatures, and the removal of hematoma from the abdominal cavity.^2,7–10)^ However, it can be insufficient or time-consuming to locate the source of bleeding due to complex anatomy and possible extensive adhesions in the surgical field. Additionally, the need to transport the patient to the operating room (OR) and prepare for general anesthesia introduces a notable delay in controlling the bleeding. If all ORs are occupied, further waiting time may be required before a laparotomy can be initiated.

Here, we present a unique case of successful management of delayed PPH, utilizing temporary balloon occlusion of the common hepatic artery (CHA) as a bridge to surgery.

## CASE PRESENTATION

**Fig. 1:**
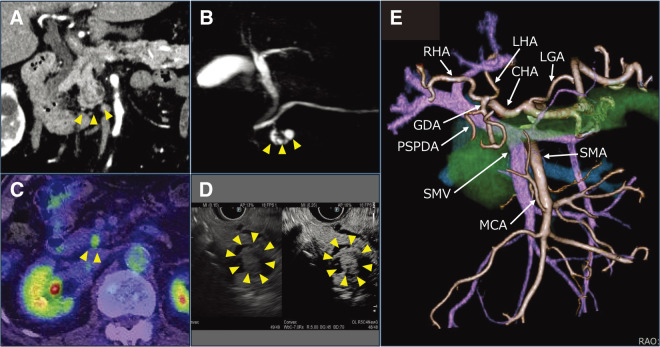
Preoperative imaging studies. (**A**) Contrast-enhanced CT image. (**B**) Magnetic resonance cholangiopancreatography imaging. (**C**) Positron emission tomography/CT image. (**D**) Endoscopic ultrasonography image. (**E**) Three-dimensional image of the main vessels and pancreas. Increased fluorodeoxyglucose uptake in the pancreatic head and enhanced mural nodule detected via endoscopic ultrasonography raised suspicion of malignancy. CT, computed tomography; RHA, right hepatic artery; LHA, left hepatic artery; CHA, common hepatic artery; LGA, left gastric artery; SMA, superior mesenteric artery; MCA, middle colic artery; SMV, superior mesenteric vein; PSPDA, posterior superior pancreaticoduodenal artery; GDA, gastroduodenal artery

A 79-year-old man with a history of pacemaker implantation for atrioventricular block was diagnosed with branch duct-type intraductal papillary mucinous neoplasm at a previous hospital (**Figs. 1A**–**1D**). Follow-up positron emission tomography/CT revealed an enhanced nodule in the cyst with fluorodeoxyglucose accumulation, raising suspicion of early pancreatic head cancer (**Fig. 1C**). He was referred to our department for treatment. Dynamic CT showed that the tumor was distant from the portal vein and did not require venous reconstruction. The proper hepatic artery (PHA) was very short, branching into the left and right hepatic arteries immediately after the gastroduodenal artery (GDA) bifurcation (**Fig. 1E**). He subsequently underwent robot-assisted pylorus-preserving pancreaticoduodenectomy. The GDA was dissected after ligation with 3-0 Vicryl sutures (Johnson & Johnson MedTech, New Brunswick, NJ, USA) and secured using a single Hem-o-lok ML clip (Teleflex, Inc.,Wayne, PA, USA). Similarly, the posterior superior pancreaticoduodenal artery (PSPDA) was managed with the same technique, involving ligation and clipping. During lymph node dissection, the nerve plexus around the hepatic arteries was preserved. Reconstruction was performed using the Child-IIA method. Choledochojejunostomy was performed using a continuous suture technique with 5-0 PDS-II sutures (Johnson & Johnson MedTech) for both the anterior and posterior walls, The operation time was 773 minutes, and the estimated blood loss was 120 mL.

**Fig. 2:**
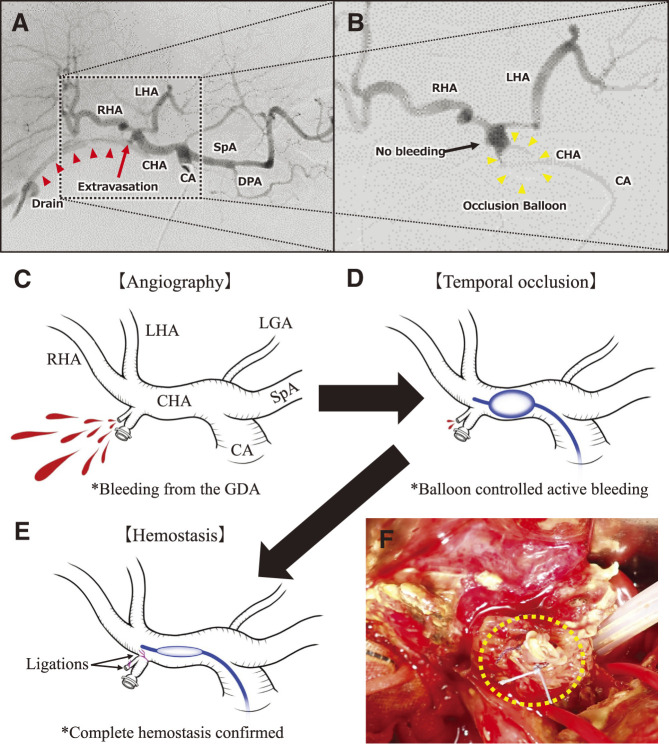
Radiological and surgical intervention. (**A**) Digital subtraction angiography image with selective catheterization of the CA. (**B**) Angiography image after temporary occlusion of the CHA. (**C–E**) Schematic representation from angiography to the completion of hemostasis. (**F**) Intraoperative photograph post-hemostasis. Active extravasation was detected near the GDA (**A, C**). Inflation of the occlusion balloon catheter placed proximal to the CHA confirmed complete occlusion of the CHA with no contrast extravasation (**B, D**). Bleeding from the PSPDA stump was visually identified by deflating the occlusion balloon. By re-ligating the PSPDA stump and the root of the GDA, we achieved secure hemostasis (**E, F**). CA, celiac axis; CHA, common hepatic artery; GDA, gastroduodenal artery; LHA, left hepatic artery; PSPDA, posterior superior pancreaticoduodenal artery; RHA, right hepatic artery; LGA, left gastric artery; SpA, splenic artery; DPA, dorsal pancreatic artery

Postoperatively, the patient developed a pancreatic fistula and bile leak, suspected to originate from the choledochojejunal anastomotic leak. On postoperative day (POD) 5, the drain discharge changed from serous to pure bile. Due to the need for a pacemaker control device and technician, surgery or endoscopic nasobiliary drainage to control the bile leakage was planned for the following day. CT imaging revealed fluid collection around the pancreaticojejunal anastomosis, leading to the conclusion that the pancreatic fistula could be managed conservatively with drain management. On POD 6, he experienced sudden bloody discharge from the abdominal drainage tubes. Hemodynamic stability was restored with intravenous fluids and blood transfusion, followed by emergent abdominal angiography. Simultaneously, preparations for emergency surgery were made, including the arrangement for pacemaker management. Abdominal aortography and selective angiography of the celiac trunk demonstrated extravasation of contrast medium from near the GDA stump (**Figs. 2A** and **2C**). A 5.2 Fr compliant latex occlusion balloon catheter, with a maximum outer diameter of 9 mm (Selecon MP Catheter II; Terumo, Tokyo, Japan), was placed proximal to the CHA and inflated (**Figs. 2B** and **2D**). The balloon was inflated at the proximal CHA due to suspected wall fragility at the bleeding site. Both the left and right hepatic arteries were visualized without any apparent issues. Active bloody drainage from the abdominal drain also stopped. No complications occurred during balloon insertion, and hemostasis remained stable.

Given the proximity of the bleeding point to the bifurcation of the CHA, coil embolization of the GDA was deemed infeasible. While coil embolization of the CHA or placement of a covered stent was considered, hepatic infarction was unavoidable with embolization. In addition, due to the very short PHA, even if stenting was successful, sacrificing the left hepatic artery (LHA) would have been unavoidable, and the risk of subsequent stent occlusion was also a concern. Additionally, the leaking choledochojejunal anastomosis, considered a cause of the bleeding, needed repair. After consultation with the radiologists and anesthesiologists, we proceeded with temporary occlusion followed by laparotomy for definitive control.

Intermittent occlusion of the CHA was maintained, with deflation every 10 minutes for approximately 3 minutes to prevent necrosis of the CHA wall, until the OR was ready. The patient was then transferred to the OR with the occlusion balloon still inflated. After thorough washing and hematoma removal, surgical exploration revealed a leak at the choledochojejunal anastomosis as the cause of bile leakage. After excising the choledochojejunal anastomosis and fully exposing the area around the GDA stump, the balloon catheter was deflated, revealing spurting bleeding from the PSPDA stump. The clip and ligation suture on the PSPDA were found to have detached during the reoperation, likely due to exposure to bile and pancreatic juice. The arterial walls of the CHA and PHA, which were covered by nerve plexus, were observed to be intact. We re-ligated the roots of the PSPDA and the GDA, achieving secure hemostasis (**Figs. 2E** and **2F**). Upon re-examination of the choledochojejunal anastomosis, the posterior wall was found to be well-sutured and intact, whereas a portion of the anterior wall appeared fragile and poorly perfused. This fragility might have resulted from robotic manipulation and potential thermal injury during the initial surgery. To address this, the bile duct wall was partially trimmed, and the anastomosis was re-sutured. Intraoperative ultrasound confirmed adequate blood flow to the entire liver. Drainage tubes were repositioned appropriately, and the surgical site was closed. The total duration of the balloon occlusion procedure, from the initial inflation in the angiography suite to its deflation in the OR, was approximately 115 minutes. The occlusion balloon was deflated after hemostasis was achieved and removed after abdominal closure. The operation time was 208 minutes, and the estimated blood loss, including hematoma, was 3000 mL.

The patient was admitted to the intensive care unit for 2 days following the reoperation with no further evidence of bleeding or bile leakage. Postoperative abdominal ultrasound confirmed good hepatic arterial blood flow. After successful conservative management of the pancreatic fistula through drainage tube management, the patient was discharged 51 days after the initial pancreaticoduodenectomy. Both clinical and laboratory follow-up findings were unremarkable. The final pathological diagnosis was intraductal papillary mucinous neoplasm with low-grade dysplasia. Eight months after the hemostasis surgery, contrast-enhanced CT revealed good blood flow from the CHA to PHA with no stenosis or aneurysm formation.

## DISCUSSION

Delayed PPH is a significant complication that necessitates prompt and effective intervention to ensure patient survival. Due to their infrequency and life-threatening urgency, constructing treatment strategies and management guidelines through randomized studies is challenging, leaving only a series of empirical reports for guidance. Historically, techniques like intra-aortic balloon occlusion, similar to those used in trauma surgery, and temporary celiac artery occlusion during angiography in critically unstable hemodynamic situations have been reported.^11–13)^ Our case represents a novel contribution to this field, utilizing a strategic approach that combines temporary balloon occlusion of the CHA with laparotomy hemostasis, thereby considering short- and long-term functionality. To the best of our knowledge, this is the first report to describe the planned and super-selective use of balloon occlusion of the hepatic arteries as a bridge to surgery, highlighting its potential as a valuable treatment strategy.

Classically, delayed PPH has been addressed through laparotomy, which includes treatment of hemorrhage causes such as anastomotic leakage and hematoma removal. However, postoperative adhesions and inflammation can complicate surgery, making it a more invasive option for critically ill patients, as blood loss continues until the bleeding site is reached. Consequently, angiography has become the recommended first line of care for PPH, particularly when hemorrhage due to a pseudoaneurysm is suspected.^14)^ Abdominal and pelvic multi-detector CT is advised for hemodynamically stable patients when sentinel bleeding is observed to identify the presence of extravasation and pseudoaneurysms.^3)^

The key point in the radiological approach is choosing between embolization and stent graft placement. Ideally, the goal is to maintain hepatic artery blood flow while achieving hemostasis. When extravasation from the GDA is confirmed, embolization is preferred if it does not compromise flow in the CHA. Endovascular covered stent placement becomes an option if the remnant GDA stump is too short for embolization or if the hemorrhage originates from the CHA to the PHA.^15)^ This technique preserves hepatic arterial blood flow while mitigating major hepatic complications such as ischemia, liver abscess, and hepatic failure. Additionally, covered stent endoprosthesis is associated with a lower risk of recurrent hemorrhage compared to selective stump embolization.^16–18)^ However, it may be challenging to perform in cases of tortuous arteries or anatomical variation, and even if successful, most stents are reported to be occluded in the long term.^19–21)^ Also, stent grafts are not always readily available in many hospitals, and there are time constraints. Hepatic artery embolization is a viable alternative for patients with complex anatomy and a high risk of recurrent hemorrhage. In cases where hepatic blood flow via other routes is anticipated, embolization may be the most reliable and fastest method of achieving hemostasis. However, it carries the potential risk of hepatic infarction, particularly if collateral branches that support hepatic blood flow were previously ligated during pancreatic surgery.

Our previous report showed that 33 patients (3.0%) out of 1096 who underwent pancreaticoduodenectomy experienced PPH.^22)^ The incidence of in-hospital and 90-day mortality relevant to PPH was one patient (3.0%) and zero patients, respectively. Although IR treatment is the first choice at our institute, we performed laparotomy in patients with delayed PPH under the following circumstances: when no imaging modality or physical examination could identify the source of bleeding; in cases of unstable hemodynamics where endoscopic or IR treatments were not feasible; when hemorrhage could not be controlled with angiography or endoscopy; for intraabdominal hematomas too large to be drained by other interventions; or when hemostasis and drainage could be safely accomplished through surgery. In the present case, angiography suggested bleeding from the GDA or one of its branches. Anatomically, the PHA immediately branched into the left and right hepatic arteries. Simply embolizing the GDA stump seemed inadequate, and endovascular artery stents would have required insertion into the CHA to the right hepatic artery, thereby covering LHA and increasing the risk of hepatic complications. Fortunately, the patient’s hemodynamics stabilized with blood transfusions, allowing a comprehensive evaluation through angiography before proceeding to the OR. Additional time was required to arrange for external clinical engineers to manage the patient’s pacemaker preoperatively, ensuring a safe surgical procedure. Intraoperatively, we capitalized on the bleeding control provided by temporary balloon occlusion, which allowed us the time needed to meticulously identify the bleeding point, secure a workspace in a hemostatic condition, and subsequently ligate it safely, resulting in a successful outcome. Since backflow was not confirmed during angiography, and no bleeding from the PSPDA was observed until balloon deflation, CHA embolization would have carried a high risk of hepatic infarction, underscoring the success of our strategy in this case. Using a temporary balloon catheter to stabilize the patient’s hemodynamics would have also been useful while awaiting the arrival of a stent graft. However, it is important to note that access to a skilled radiologist for IR, as well as the availability of emergency surgery with sufficient anesthesiologists and surgical staff members, is not always guaranteed. Even in high-volume centers, managing such situations can be challenging, particularly depending on the timing. We were fortunate this time to have the right conditions available. Additionally, we must reaffirm that pancreaticoduodenectomy is a technically demanding surgery requiring perioperative support from multi-specialists.

## CONCLUSIONS

We report a strategic combination of IR and surgery. In hemodynamically stable cases, this strategy may contribute to achieving adequate hemostasis, avoiding short-term liver complications, and maintaining long-term hepatic functionality.

## DECLARATIONS

### Funding

No funding was received for this study.

### Availability of data and materials

All data generated during this report are included in this published article.

### Ethics approval and consent to participate

This work does not require ethical considerations or approval.

### Consent for publication

Written informed consent was obtained from the patient for publication of this case report and any accompanying images.

### Competing interests

The authors declare that they have no competing interests.
